# Social networks, mobility, and political participation: The potential for women’s self-help groups to improve access and use of public entitlement schemes in India

**DOI:** 10.1016/j.worlddev.2018.09.023

**Published:** 2019-02

**Authors:** Neha Kumar, Kalyani Raghunathan, Alejandra Arrieta, Amir Jilani, Suman Chakrabarti, Purnima Menon, Agnes R. Quisumbing

**Affiliations:** a International Food Policy Research Institute, Washington, DC, United States; b International Food Policy Research Institute, New Delhi, India

**Keywords:** Self-help groups, India, Entitlements, Women, Social networks, Political participation

## Abstract

Women’s self-help groups (SHGs) have increasingly been used as a vehicle for social, political, and economic empowerment as well as a platform for service delivery. Although a growing body of literature shows evidence of positive impacts of SHGs on various measures of empowerment, our understanding of ways in which SHGs improve awareness and use of public services is limited. To fill this knowledge gap, this paper first examines how SHG membership is associated with political participation, awareness, and use of government entitlement schemes. It further examines the effect of SHG membership on various measures of social networks and mobility. Using data collected in 2015 across five Indian states and matching methods to correct for endogeneity of SHG membership, we find that SHG members are more politically engaged. We also find that SHG members are not only more likely to know of certain public entitlements than non-members, they are significantly more likely to avail of a greater number of public entitlement schemes. Additionally, SHG members have wider social networks and greater mobility as compared to non-members. Our results suggest that SHGs have the potential to increase their members’ ability to hold public entities accountable and demand what is rightfully theirs. An important insight, however, is that the SHGs themselves cannot be expected to increase knowledge of public entitlement schemes in absence of a deliberate effort to do so by an external agency.

## Highlights

• Our paper explores the link between women's self-help groups and public entitlements.
• It further examines the effect of SHG membership on social networks and mobility.
• Empirical strategy employs matching methods to correct endogeneity of SHG membership.
• Using data from rural India, we find that SHG members are more politically engaged.
• SHG members are more likely to know of certain public entitlements than non-members.
• SHG members are also more likely to avail of these public entitlement schemes.
• SHG members also have wider social networks and greater mobility than non-members.

## 1 Introduction

Women’s groups have increasingly been used as a vehicle for social, political, and economic empowerment. Although they can be found in various forms all over the world (Meinzen-Dick, Behrman, Pandolfelli, Peterman, & Quisumbing, 2014), self-help groups (SHGs) are most visible in India, where they have been facilitated by NGOs, the government, and even the private sector (Desai & Joshi, 2014). SHGs are “membership-based organizations” whose members provide each other with mutual support while attempting to achieve individual objectives through access to savings and loans and linkages to banks (Bouman, 1995, Shah et al., 2007, Tankha, 2002), as well as collective objectives through community action (Chen, Jhabvala, Kanbur, & Richards, 2006 cited in Desai & Joshi 2014). Each SHG typically consists of 10–20 poor women from similar socio-economic backgrounds who live near each other, meet regularly, and save small amounts of money in a common account. SHGs were originally founded to provide access to savings and credit to women who were outside the reach of the formal banking sector (Tankha, 2002, Swain, 2006, Swain and Wallentin, 2012). However, these groups are increasingly being leveraged by government and non-governmental organizations as a platform for reaching communities to strengthen rural livelihoods, improve women’s empowerment and agency, increase demand for – and accountability of - public entitlements, and deliver information on health and nutrition.

In this paper, we examine the potential for women’s SHGs to improve access to and use of public entitlement schemes. Access to and use of such schemes involves both supply- and demand-side constraints. In this paper, we consider demand side constraints and how SHGs can alleviate them.^1^ These constraints include *information* about the schemes among potential beneficiaries to take advantage of the schemes and the *ability of potential beneficiaries to hold the public entities accountable*. Insights from related work (Kumar et al., 2017) may shed light on the pathways through which SHGs may influence women’s access to and use of public entitlement schemes. Kumar et al. (2017) propose a conceptual framework that outlines the pathways through which women’s groups may facilitate improvements in nutrition. The four main pathways involve: (1) the generation of income through savings and credit, (2) improvement of agriculture and livelihoods through engagement of women, (3) nutrition-related behavior change communication, (4) the rights pathway, which involves training SHG members in accountability. In addition, three cross cutting themes – building social capital, acting collectively and promoting women’s empowerment – are identified as core components of each pathway.

Of these, the rights and social accountability pathway along with the cross-cutting themes may be relevant for increased awareness and use of publicly provided entitlement schemes.^2^ The rights pathway is relevant to increased use and awareness of public entitlements if the women’s group promotes awareness and use of specific health and nutrition related programs, through a combination of increased demand and coordination with service providers. There may be a direct link from SHGs to increased awareness and use of public entitlement schemes if the organizing institution has a mandate to increase awareness and utilization of certain public entitlement schemes within their SHGs.

Even without this mandate, government organizations and NGOs that support the formation of women’s SHGs indirectly help create larger networks and greater communication within those networks, owing to the intrinsic modality of the groups. Greater communication can lead to greater flow of information. Women could learn about public entitlement schemes from their group members even if the group is not organized with an explicit objective to increase awareness about public entitlements.

A second channel to increased information may be via increased mobility among women in SHGs. To attend the group meetings, the women need to leave their homestead, increasing their mobility (albeit within their own village). Women that are part of SHGs, because of regular interactions not only with group members but also with external agents that facilitate these groups, may become more adept at communicating. Often, this experience of being in a group and interacting with other women can in itself boost women’s self-confidence. Family members (mostly husbands) of women in SHGs may also feel more confident about the ability of these women to leave the homestead and engage with the outside world, perhaps even going outside the village (with their group).

The ability to hold public entities accountable, the second demand side constraint identified above, is more complex. One can view this as a culmination of factors - improved social networks, greater mobility and greater self-confidence – that may lead to greater political participation, which in turn may lead to greater accountability. Women in SHGs meet regularly for their group meetings, which exposes them to the practice of meeting in groups and may make them more likely to attend village council meetings (the *gram sabha* and the *mahila gram sabha*, described in Section 5). The collective voice of the group, along with increased self-confidence, gives them further encouragement to raise issues at these meetings and demand their rights. Drawing on these conceptual underpinnings, we examine whether SHG membership increases political participation, awareness, and utilization of public entitlements among its members. We enrich this analysis by examining whether SHG membership leads to increased social networks, self-confidence and mobility.

To assess the impact of SHG membership on political participation and awareness and use of government entitlements, this paper draws on cross-sectional data collected in 2015. The data used here is from the baseline survey of an evaluation of the impacts of layering nutrition-sensitive interventions, including those that foster greater awareness and use of government health, nutrition and food security programs, on an NGO’s existing agricultural-livelihoods program platform. We are constrained by the cross-sectional nature of our data and the fact that SHGs were already functional in the study areas before the baseline was conducted, but we attempt to correct for the endogeneity of SHG membership using nearest-neighbor matching estimators. We find that, compared to non-SHG members, SHG members are more likely to know and interact with other women, even those outside their locality, are more likely to vote, and to vote according to their own choice, and are more likely to attend village meetings. SHG members are not only significantly more likely to know about certain public entitlements, particularly those that are targeted to the household, but are also more likely to avail of a greater number of public entitlement programs. We argue that, while knowledge about these public entitlements may be widespread, even among non-SHG members, SHG members may feel more empowered to assert their rights and avail of these entitlements.

The paper is organized as follows. Section 2 provides a brief review of related literature and background on SHGs and government programs in our study area. Section 3 describes the data and presents descriptive statistics about the sample. Section 4 discusses the methods used in the paper, while Section 5 presents the results on the impact of SHG membership on outcomes related to social capital, political participation, and awareness and use of government entitlement programs. Section 6 concludes.

## 2 Related literature and context

### 2.1 Related literature

Brody et al. (2017) review the literature on the impact of economic SHGs on women’s empowerment, and hypothesize pathways through which SHGs may empower women. They discuss how access to resources (such as credit and training), exposure to group support and accumulation of social capital can, in the long term, lead to positive economic, political, social or psychological empowerment of women. Overall, their review of the literature suggests that SHGs can have positive effects on women’s economic, political and social empowerment, but they emphasize the need for more rigorous quantitative analyses. Our paper adds to the body of evidence on the effectiveness of SHGs in improving these outcomes (Deininger and Liu, 2009, Deininger and Liu, 2013a, Deininger and Liu, 2013b, Swain and Kumaran, 2012, Desai and Joshi, 2014; and papers cited in Brody et al., 2017) by studying the association of SHG membership with improved political participation, social capital, and the awareness and utilization of government schemes.

The four quantitative studies included in (Brody et al., 2017)’s meta-analysis that examined the impact of SHGs on political empowerment varied considerably both in terms of evaluation design and the degree of attention paid to the measurement of political empowerment and governance. Only (Desai & Joshi, 2014), who worked with the Self-Employed Women’s Association (SEWA) to randomly assign an SHG program to treatment communities, used an experimental design; (Pitt, Khandker, & Cartwright, 2006) and (Deininger & Liu, 2013a) used a quasi-experimental design, and (Swendeman, Basu, Das, Jana, & Rotheram-Borus, 2009) calculated risk or odds ratios based on events/non-events.

The measures used to capture political participation range from relatively simple indicators, such as voting behavior, to more comprehensive and sophisticated measures of political engagement. (Deininger & Liu, 2013a)’s evaluation of the impacts of an SHG-delivered micro-credit program in Andhra Pradesh used attendance at meetings and trust in village officials as indicators of political participation and social capital. (Pitt et al., 2006)’s quasi-experimental study examined responses to a range of questions related to political activism, awareness of law and politics, and autonomous action on public and private matters, which were combined into a single factor. Finally, (Desai & Joshi, 2014) used comprehensive measures of civic engagement in their experimental study that randomized the establishment of SHGs across villages. They measured respondents’ knowledge of where to report grievances relating to problems with water/sanitation, poor road conditions, faulty electricity supply, and inadequate education and health services, and also measured whether the respondent actually approached authorities to report a complaint and demand improvements in delivery. They also examined women’s awareness of bribes being collected from villagers, and their participation in the main local government institutions, the *gram sabha* and the *gram panchayat -* village meetings that form the foundation of the decentralized village governance system known as the *Panchayati Raj*.

The quantitative evidence on the association between membership in women’s groups and political participation is limited but largely positive. (Swendeman et al., 2009)’s study of an intervention to empower sex workers found that political participation, measured as voting, did not improve significantly, although the empowerment intervention may have prevented coerced voting. (Deininger & Liu, 2013a) found that 6% of women attended village meetings (*gram sabha*) more frequently because of the intervention, an SHG-delivered micro-credit program, and that the program contributed to an estimated increase of trust in other villages, elected representatives, or government representatives of between 5% and 15% points. (Pitt et al., 2006) showed that credit extended to women positively affected the factor relating to women’s awareness and activism, the odds that a woman was informed about the ways that a premarital bridal contract can be used to help a woman in case of divorce, the probability that a woman knew the name of the member of parliament in her area, that she voted in the last election^3^, and that she voted independently (rather than upon the advice of her husband). In contrast, male-targeted credit reduced the probability that his wife claimed to have voted independently. Finally, (Desai & Joshi, 2014) found that women in SHGs were more likely to know where to report grievances regarding water, and were also more likely to have reported these grievances.^4^

In this paper, we measure political participation using indicators of whether the respondent voted in the last election, and made the decision to vote without coercion from family members or others, as well as whether she participated in the *gram sabha* or the *mahila* (women’s) *gram sabha*. In addition, we study the awareness and utilization of a range of government entitlement schemes targeted at households, and at women and children. Though the studies discussed above did not look explicitly at awareness and use of government entitlement schemes, it is likely that the same mechanisms that increase women’s political empowerment could also operate to increase their knowledge of their entitlements and their claim on the benefits due to them. For example, by disseminating information about local institutions, governmental programs, policies, and procedures, SHGs may lower the cost of accessing information about community issues (Desai & Joshi, 2014). (Desai & Joshi, 2014) also show that there is evidence that women in SHGs are more likely to know where to report grievances related to various public services and to also report grievances. The group meetings and social networks facilitated by SHGs make it easier to disseminate information as well as to deliver services; instead of going to individual women’s homes to deliver messages about livelihoods, credit, health and nutrition, for example, extension workers from relevant government departments or from NGOs could save time and money by using group meetings, typically at a more centrally located place.

In addition to political empowerment, (Brody et al., 2017) also synthesizes the evidence around the impact of SHGs on women’s social empowerment, as measured by increased mobility, improved decision-making power within the household (particularly around family-size), increased challenge of gender norms, and the use of contraceptives. The studies are located in varied geographical contexts, though much of the evidence is concentrated in South Asia. While the results of the three RCTs included in the meta-analysis are somewhat inconclusive, with positive but often insignificant effect sizes, the quasi-experimental studies included in the review show a positive and significant impact of group participation on social empowerment measures. In our paper, we measure social capital by the size and quality of the respondent’s social network (‘quality’ measured by conversational contact, as well as the ability to borrow from within one’s network), and also by her mobility and her ability to speak out in public.

Shankar and Gaiha (2012) show that political networks and social networks are important correlates of knowledge of decision making around a public workfare scheme, the Mahatma Gandhi National Rural Employment Guarantee Act (MGNREGA). They note that households that were only socially networked were no more likely to be aware of these decisions compared to households that were not socially networked. This underscores the importance of being politically networked in addition to being socially networked to be better aware of public schemes. In addition, there is evidence on the use of health services and the role that SHGs may play in facilitating uptake. For example, using a cross-sectional dataset from India, Saha, Annear, & Pathak (2013) find that presence of SHGs in the village was positively associated with knowledge of family planning and use of health services.

In addition to the quantitative studies cited above and in Brody et al. 2017, a large body of qualitative work studies the impact of Indian SHGs on several of our outcomes of interest. Sanyal (2014) uses very rich and detailed qualitative data to highlight the impact of microfinance groups on women’s agency, on the creation of social capital, and on women’s ability to act collectively to demand public goods and resolve issues that go beyond individual needs. Using data from West Bengal, Sanyal (2009) finds that individual empowerment fosters social cooperation, allowing women to act collectively towards goals that benefit those outside the group as well. For example, women collectively rallied around issues of public goods, such as the provision of sanitation facilities and the repairing or installation of pathways. They conclude that rather than limiting cooperation, the regularity and interest that the group’s economic transactions provide seem to facilitate social cooperation. Davidson and Sanyal (2017) echo this finding using data from Karnataka. They find that, in comparison to non-SHG women, SHG members form significantly greater number of ties with people they are not related to, and that these relationships derive from the SHG ties. Given low levels of non-kin ties among women in the rural Indian context, this is a significant outcome.

In the context of a larger quantitative evaluation of the JEEViKA program in Bihar, Sanyal, Rao and Majumdar (2015) conducted in-depth qualitative studies in four villages, two treatment and two control villages. They found that SHG women exhibited far greater mobility than their non-SHG counterparts. Part of the increased mobility was induced through group meetings held outside the home, but women slowly began to challenge norms and lay claim to public spaces that were traditionally reserved for men. SHG women also regularly attended and participated in public meetings, and arbitrated on behalf of others on issues such as domestic violence. Through JEEViKA, women engaged in making eye contact and introductions and public speaking, all of which served to increase their confidence. This increased confidence in public seems to be common to multiple SHG contexts. For example, Sanyal, Rao, and Prabhakar (2015) analyzed the transcripts of more than 250 *gram sabha* meetings in four southern states, and found that SHG women employed a greater number of narrative styles and were able to convey their demands or complaints more convincingly to public officials. Finally, Sanyal, Rao, and Prabhakar (2015) also provide evidence that while the range of issues SHG and non-SHG women raised were often similar, issues that affected the community at large were more likely to have been raised by SHG women. The qualitative evidence supports the findings of the quantitative studies, and both document how the impacts of belonging to an SHG go well beyond direct economic benefits; conferring on women greater personal freedoms and a larger role in shaping their own communities.

### 2.2 Context

In the 1980s, SHGs in India, sought to reduce poverty and improve livelihoods in poor, rural communities. Early government initiatives focused on addressing credit constraints by linking SHGs to banks (Shah et al., 2007, Tankha, 2002), and microcredit for poverty reduction was the basis of the key national SHG programme, Swarnajayanti Gram Swarojgar Yojana (SGSY), which was implemented under the Ministry of Rural Development (MoRD) from 1999 to 2011 (extended to 2013) (OPM 2014). Over the last few decades, SHG programs, particularly at the state level, have expanded to include efforts promoting social mobilization, social accountability, awareness of rights and entitlements and more recently, targeted programming to improve health and nutrition. Among these are SERP (the Society for Elimination of Rural Poverty) in Andhra Pradesh, which is linked to the Indira Kranti Patham (IKP) programme, JEEViKA in Bihar and Kudumbashree in Kerala. Eventually, the National Rural Livelihoods Mission (NRLM) was launched as the Government of India’s replacement for SGSY in 2011 (and re-launched in 2013) and is heavily influenced by the State level programmes such as SERP.^5^

SHGs, also known as mutual aid or support groups, are small voluntary groups that are formed by people related by an affinity for a specific purpose who provide support for each other (Brody et al., 2017). SHG members use strategies such as savings, credit, or social involvement as instruments of individual and collective empowerment.

The standard economic SHG model starts with an initial period of collective saving. A typical SHG has anywhere between 10 and 15 female members who meet once a week. Each week each woman deposits a small amount, typically INR 10^6^, in a common box that forms the group’s collective savings, from where members can borrow money. Groups of SHGs are federated into higher level platforms that differ somewhat from location to location, the most common being the Village Level Federation or Village Organization that consists of all women from three to five SHGs in the village. Chosen or appointed representatives from each SHG attend the higher-level Federation meetings, and represent their group’s interests at those gatherings. In addition to the savings in the common fund, SHG meetings are used to discuss matters of interest to the group, disseminate information regarding health, nutrition and livelihoods, and plan community-led events.

While SHGs are formed primarily to encourage group-level savings and credit systems, they often become vehicles for social change along several different dimensions, e.g. agriculture and livelihoods, gender, rights and entitlements, and (more recently) health and nutrition.^7^ Most organizations forming these groups take the somewhat nebulous concept of improved ‘women’s empowerment’ as a key outcome of the process of collectivization. Women’s empowerment is measured in a variety of ways – increased mobility both within and outside the village, increased political awareness and participation, especially in local governmental bodies, increased participation in decision-making within the household around purchases and livelihoods, among others.

In this paper, we focus on a subset of these outcomes, notably political participation, improved awareness and utilization of government entitlement schemes, and some mechanisms – mobility, social networks – that could potentially help explain those outcomes. Table 1 summarizes the eligibility criteria and the benefits under the various government entitlement schemes. These schemes have been divided into those available at the household-level and those that are targeted toward women and children within the 1000-day window between conception and two years of age. The table shows that eligibility criteria and benefits of the schemes vary substantially.

**Table 1:** Eligibility criteria and benefits of government entitlement schemes.

Scheme	Eligibility criteria	Benefits	Source
*Schemes targeted to households*			
Mahatma Gandhi National Rural Employment Guarantee Act (MGNREGA)	• Persons 18+ years from households in rural areas (except Jammu and Kashmir) • Households must have a job card	100 days of unskilled manual labor at a pre-specified state minimum wage	http://nrega.nic.in/amendments_2005_2016.pdf
Indira Awas Yojana (IAY)	• BPL households, especially those identified as needy by the *gram sabha* • House must include toilet, smoke pit, compost pit and smokeless *chulhas*	Financial assistance in • the construction of new homes (especially for the homeless) • the upgradation of *kuccha* (impermanent) or dilapidated homes	http://iay.nic.in/netiay/home.aspx
Public Distribution System (PDS)	• Households that have an AAY, BPL or APL card	5 kg of rice/wheat/coarse grain per person at prices of Rs. 3/2/1 respectively	http://www.pdsportal.nic.in/files/PDS(Control)%20Order,%202015.pdf
Antyodaya Anna Yojana (AAY)	• Poorest of the BPL category • Households must have a BPL card as well as an Antyodaya Ration card	35 kg of rice/wheat/coarse grain per household per month at prices of Rs. 3/2/1 respectively	http://www.pradhanmantriyojana.co.in/antyodaya-anna-yojana/
*Schemes affecting mothers and children in the 1000 day window*			
Integrated Child Development Scheme (ICDS)	• Children aged 0–6 years • Pregnant and lactating women	• Supplementary nutrition • Immunization • Health check-ups • Referral services	http://icds-wcd.nic.in/icds/icds.aspx
	• Children aged 3–6 years	• Pre-school education	
	• Women aged 15–45 years	• Nutrition and health education	
Janani Suraksha Yojana (JSY)	All pregnant women belonging to BPL (Below Poverty Line) households • of the age of 19 years or above, and • for up to two live births, • provided the child is born in a health institution. • Benefits will be extended to the third birth for women form BPL households in 10 low performing states, provided they elected to undergo sterilization immediately after delivery.	Low performing states: • Rural areas: Rs. 1400 • Urban areas: Rs. 1000 High performing states: • Rural areas: Rs. 700 • Urban areas: Rs. 600 Urban area: NIL	http://www.nhp.gov.in/janani-suraksha-yojana-jsy-_pg
Janani-Shishu Suraksha Karyakram (JSSK)	• All pregnant women • All newborn children • Sick infants up to 30 days	• Free-of-charge delivery in a government institution • Free transport to and from home to the government institution Free drugs, diagnostic tests, food etc	http://jknrhm.com/guidelines_for_jssk.pdf

Schemes targeted at households are sometimes restricted to those households with a BPL (Below Poverty Line) card, as with the financial assistance for the construction of houses (the Indira Awas Yojana). Other schemes like the Public Distribution Scheme have different entitlements of foodgrains for households of different degrees of poverty. Finally, workfare schemes like the Mahatma Gandhi National Rural Employment Guarantee Act (MGNREGA) are self-targeting, and are not restricted to any particular income group.

Among the schemes aimed at pregnant and lactating mothers and young children, the Integrated Child Development Scheme (ICDS) is the oldest, dating back to 1975. This scheme provides supplementary nutrition to mothers and children through local ICDS centers. In addition, the ICDS performs the role of a crèche, providing pre-school education to children aged 3–6 years. The Janani Suraksha Yojana (JSY), introduced in 2005, is a scheme aimed at improving childbirth in an institutional setting. Mothers and frontline health and ICDS workers are provided financial incentives to deliver the child in a health institution, with the amounts varying both within and across states. Finally, the Janani-Shishu Suraksha Karyakram (JSSK) ensures free-of-cost medical care to pregnant women and newborn children.

## 3 Data and descriptive statistics

### 3.1 Data

This study draws on data from a baseline survey conducted from September to December 2015 in eight districts of five states of eastern and central India – Madhya Pradesh, Orissa, Chhattisgarh, Jharkhand and West Bengal. The baseline survey forms the first wave of an impact evaluation of nutrition-intensification efforts being made by an Indian NGO, PRADAN. Three blocks were selected in each district of the study, making a total of 24 blocks. From each of the blocks between five and seven villages were chosen at random from the full list of villages, and from each village 20 women were selected at random from among all ever-married women aged 15–49. The final sample size at baseline was 2744 women. Sample selection was not conditioned on SHG membership, and at baseline approximately 38% of our sample belonged to an SHG. The low level of saturation allows us to compare outcomes across women who belong to an SHG and those who do not.

Women’s SHGs in our area of study could be formed by PRADAN, by other NGOs, or by the government under NRLM. Unfortunately, we do not have information in our baseline survey on which organizations, governmental or otherwise, form and support these SHGs; in most cases, our respondents were not able to identify which organization supported the SHG to which they belonged.^8^ For this study, therefore, we treat all SHGs as being broadly similar in their functioning.

### 3.2 Descriptive statistics

#### 3.2.1 Demographic and socio-economic characteristics

SHG members are, on average, about 2.6 years older than non-members and have been married for about 2.9 years longer (Table 2). The mean length of SHG membership was 4.2 years at baseline. SHG members are less likely to self-identify as housewives, and more likely to have bank accounts – approximately 59 percent of members had a bank account, compared to 42 percent among non-SHG members. Differences between members and non-members in caste composition and women’s education are not significant.

**Table 2:** Summary statistics, by SHG membership.

Variable	SHG Membership			Difference in means
N = 2744	Full sample	Members	Non-members	Members vs Non-members
Age of women respondent	32.886	34.503	31.883	2.620***
Woman has 1–5 years of schooling	0.149	0.158	0.144	0.014
Woman has more than 5 years of schooling	0.199	0.184	0.208	−0.024
Ag & Non-Ag day Laborer	0.366	0.390	0.352	0.038
Housewife	0.263	0.222	0.289	−0.067***
Caste of household head, SC	0.120	0.130	0.113	0.016
Caste of household head, ST	0.668	0.639	0.687	−0.047
Caste of household head, OBC	0.165	0.179	0.157	0.022
Married	0.925	0.930	0.922	0.008
# years married+	15.654	17.486	14.518	2.968***
Dummy for whether the husband of respondent is present in HH	0.877	0.881	0.875	0.006
Attitude towards gender equity normalized+	0.717	0.729	0.710	0.018
Has own money to use	0.440	0.460	0.428	0.032
Talk often to own family other than HH*	0.547	0.535	0.555	−0.020
Leisure hours per day	8.803	8.768	8.825	−0.057
Work hours per day	4.991	5.271	4.817	0.454***
# of children under 5 years	0.567	0.514	0.600	−0.085*
No. females age 10–55 years	1.668	1.701	1.647	0.054
Has bank account	0.483	0.586	0.420	0.166***
Ability to borrow from multiple sources*	0.430	0.448	0.420	0.028
Social status weight*	0.216	0.217	0.215	0.002
Sum of 4 locus of control questions (range 4–16)*	10.625	10.686	10.587	0.098
Sum of 4 self-esteem questions (range 4–16)*	10.866	10.861	10.870	−0.009
Sum of 4 trust questions (range 4–16)*	10.544	10.625	10.494	0.131
Per capita monthly total expenditure (in INR)	770.03	755.97	778.74	22.77
Wealth index	0.000	0.185	−0.115	0.299**
Poorest wealth quintile	0.200	0.164	0.223	−0.059***
No. of types of assets woman owns+	4.057	4.235	3.947	0.288**
HH owns more land than average in that district	0.331	0.349	0.320	0.029*
HH owns more large livestock than average in that district	0.419	0.468	0.389	0.079***
HH owns more small livestock than average in that district	0.210	0.245	0.189	0.056**
Women's average education per village	2.298	2.336	2.275	0.061
Range of highest and lowest wealth index in village	4.757	4.753	4.760	−0.006
Average land owned by HH in village (acres)	1.926	1.975	1.896	0.079
Avg. number of large livestock owned by HH in village	1.967	2.039	1.923	0.116
Avg. number of small livestock owned by HH in village	1.284	1.411	1.205	0.206**

Note: 'Social status weight' refers to the weight (proportion of beans out of 20) assigned to 'Social Status'. 'Locus of control questions' aggregate answers to 4 statements on control over their lives indicating the degree to which respondent agrees (4 indicates strongly agree). 'Self esteem questions' aggregate answers to 4 statements on self-esteem indicating the degree to which respondent agrees. 'Trust questions' aggregate answers to 4 statements on trust indicating the degree to which respondent agrees. 'Talk often to own family' refers to talking to somebody from her family at least several times per month'. 'HH Ability to borrow from multiple sources' include ability to borrow cash/in-kind from NGOs, informal lenders, formal lenders, and/or friends/relatives. '# years married', N = 2735; 'Attitude towards gender equity normalized', N = 2525; 'No. of types of assets woman owns', N = 2718. ***Indicates significant difference at p < 0.01, ** at p < 0.05, and * at p < 0.10.

There appear to be some significant differences between SHG members and non-members in terms of wealth and asset ownership. A principal components analysis of wealth that captures home, animal and mobile phone ownership, along with dwelling characteristics, availability of electricity and food security, indicates that SHG members are better-off than non-members, and that a smaller proportion of SHG members fall in the poorest wealth quintile compared to non-SHG members (16.4% versus 22.3%). SHG members are also likely to own a greater mix of assets (land, livestock, farm equipment, cell phone, etc.) compared to non-SHG members, and on average, more likely to report that their household owns more land and livestock than the average in their district. It is hard to establish the causality between SHG membership and wealth. SHG members could be better-off because of their participation in the group, but conversely, better-off women may be more likely to participate in SHGs, because they have the time or adequate resources for regular savings, for example (see Brody et al. (2017) for a discussion on exclusion from SHGs).

#### 3.2.2 Political participation

We measure the extent of political participation of the women in our sample by their previous voting behavior and attendance of *gram sabha* meetings. Table 3 provides more details on the definitions of these political participation variables. G*ram sabhas* are public meetings where villagers make important decisions about budgetary allocations for village development and the selection of beneficiaries for anti-poverty programs (Rao & Sanyal, 2010). In addition to these meetings, which can be attended by anyone in the village, some states have begun *mahila gram sabhas,* or *gram sabhas* where only women participate (*mahila* means ‘woman’). Since women’s participation (including attendance) was observed to be low in the regular village meetings, which tend to be male-dominated spaces (see Parthsarathy et al (2017)), these ‘women-only’ *gram sabhas* were established to provide women with an opportunity to voice their grievances. In the overall sample, 87.4 percent of the respondent women had a voter ID and 86.7 percent of them voted in the last election (Table 4). These numbers are considerably higher when compared to participation in village meetings – less than 10 percent of women in the whole sample ever participated in the *mahila gram sabha* (adult women’s village meeting) or the *gram sabha*.^9^

**Table 3:** Definitions of outcome variables.

Variable	Definition
*Political participation outcomes*	
Respondent women voted in the last election	1 if the respondent voted in the last election, 0 otherwise
Respondent women voted because it is her right to vote	1 if the respondent voted because it was her right to do so, 0 otherwise
Respondent women voted and made this decision herself	1 if the respondent voted and made this decision herself, 0 otherwise
Respondent women has ever participated in *mahila gram sabha*	1 if the respondent participated in the *mahila gram sabha,* 0 otherwise
Respondent women has ever participated in *gram sabha*	1 if the respondent participated in the *gram sabha*, 0 otherwise
Respondent woman believes that GP will take positive action to her demands	={1 if the respondent woman believed that the *gram panchayat* will take action in response to complaints/suggestions raised collectively by women/SHGs,
	-1 if the respondent woman believed that the *gram panchayat* will never take action in response to complaints/suggestions raised collectively by women/SHGs, 0 if the respondent does not know how the *gram panchayat* would respond}
Political participation score	(Sum of all political participation indicators)/6
*Awareness and utilization of government schemes*	
Household aware of {MGNREGA, IAY, AAY, ICDS, JSY, JSSK}	1 if respondent is aware of the scheme, 0 otherwise
Household used {MGNREGA, IAY, AAY, ICDS, JSY, JSSK}	1 if respondent has used the scheme, 0 otherwise
*Social network outcomes*	
Know at least 1/5 women	1 if respondent knows at least 1 out of 5 randomly selected women from the village, 0 otherwise
Part of social group with at least 1/5 women	1 if respondent is in a social group with at least 1 out of 5 randomly selected women from the village, 0 otherwise
Spoke to at least 1/5 women in last 5 months	1 if respondent has spoken to at least 1 out of 5 randomly selected women from the village, 0 otherwise
Social network score	(Sum of all social network indicators)/7
Spoke >9 people in the last 30 days in hamlet	1 if respondent spoke to more than 9 people in the last 30 days in the hamlet, 0 otherwise
Spoke >9 people in the last 30 days nearest hamlet	1 if respondent spoke to more than 9 people in the last 30 days in the hamlet, 0 otherwise
Could borrow 1000 rupees from at least 10 people within hamlet/village	1 if respondent can borrow 1000 rupees from at least 10 people within hamlet/village, 0 otherwise
Could borrow 1000 rupees from at least 10 people in the closest hamlet/village	1 if respondent can borrow 1000 rupees from at least 10 people in the closest hamlet/village, 0 otherwise
*Mobility and Confidence in public spaces*	
Does not need permission to go to at least one place	1 if the respondent does not need permission to go to 1 out of the 7 places identified (such as the market, friends/family’s house, place of worship, public village meeting, meeting of an association, outside the village and health care provider), 0 otherwise
Does not need permission to go to a village meeting or meeting of an association	1 if the respondent does not need permission to go to a village meeting or meeting of an association, 0 otherwise
Comfortable in speaking in public	1 if the respondent is comfortable in speaking in public, 0 otherwise
Mobility and confidence score	(Sum of all indicators of mobility and confidence)/3

Notes: All questions related to voting behavior, awareness and use of government schemes, social networks, mobility and confidence were asked after the respondent woman enrolled in an SHG.

**Table 4:** Summary statistics: political participation by SHG membership.

Variable		SHG Membership		Difference in Means
N = 2744	Full sample	Members	Non-Members	Members vs Non-Members
Respondent women voted in the last election	0.867	0.935	0.825	0.111***
Respondent women voted because it is her right to vote	0.260	0.299	0.236	0.064***
Respondent women voted and made this decision herself	0.390	0.445	0.355	0.089***
Respondent women has ever participated in *mahila gram sabha*	0.092	0.168	0.045	0.122***
Respondent women has ever participated in *gram sabha*	0.067	0.124	0.032	0.092***
Respondent woman believes that GP will take positive action to her demands	0.105	0.162	0.070	0.092***
Political participation score (range: 0–1)	0.305	0.367	0.267	0.99***

Notes: ***Indicates significant difference at p < 0.01, ** at p < 0.05, and * at p < 0.10.

We find that SHG members are in general more politically active than non-members. Almost 94% of SHG members voted in the last election, as compared to only 82.5% of non-SHG members (unadjusted p < 0.01), and a significantly higher proportion of SHG members, reportedly, made the decision to vote on their own.^10^ The difference is especially meaningful in women’s participation in the village meetings. On average, 16.8 percent of SHG members ever participated in a *mahila gram sabha* compared to 4.5 percent of non-members. Similarly, 12.4 percent of SHG members ever participated in the *gram sabha*, significantly higher than the 3.2 percent of non-members. The political participation score is higher among SHG members by about 10 percentage points and this difference is statistically significant.

#### 3.2.3 Awareness and use of government entitlements

Awareness of entitlement schemes varies in our sample (Table 5). About 78% of women have heard of the PDS, and slightly over 70% of women have heard of the MGNREGA schemes. However, less than two-thirds of the women have heard of any of the other schemes, with awareness of JSSK being the lowest at 8.2%. Compared to non-SHG members, SHG women are more likely to have heard of MGNREGA (81% versus 69%, unadjusted p < 0.01), and of IAY (72% versus 62%, unadjusted p < 0.01).

**Table 5:** Summary statistics, awareness of public entitlement programs by SHG membership.

Variable		SHG Membership		Difference in Means
N = 2744	Full sample	Members	Non-Members	Members vs Non-Members
Respondent woman is aware of MGNREGA	0.736	0.810	0.690	0.119***
Respondent woman is aware of IAY+	0.660	0.719	0.623	0.097***
Respondent woman is aware of PDS	0.783	0.818	0.762	0.057***
Respondent woman is aware of AYY	0.417	0.450	0.397	0.053***
Respondent woman is aware of ICDS	0.656	0.678	0.642	0.036
Respondent woman is aware of JSY	0.544	0.574	0.525	0.049*
Respondent woman is aware of JSSK	0.082	0.084	0.081	0.002

Note: 'Respondent women is aware of IAY', N = 2675. ***Indicates significant difference at p < 0.01, ** at p < 0.05, and * at p < 0.10.

Although several schemes have been in place for decades, overall utilization of entitlement programs is very low (Table 6). Only 34.6 percent of women used ICDS, 17.9 percent used JSY and 3.1 percent used JSSK^11^. We found no significant differences in utilization of these programs between SHG members and non-members. Utilization is higher for household-level programs, with around 45 percent of all women reporting ever having used MGNREGA and PDS. In all household-targeted programs, SHG members have significantly higher use of the public entitlement programs, on average, than non-members.

**Table 6:** Summary statistics, utilization of public entitlement programs by SHG membership.

Variable		SHG Membership		Difference in Means
N = 2744	Full sample	Members	Non-Members	Members vs Non-Members
Household used MGNREGA	0.457	0.553	0.397	0.156***
Household used IAY+	0.145	0.181	0.123	0.058**
Household used PDS	0.479	0.559	0.429	0.130***
Household used AYY	0.162	0.186	0.148	0.038**
Household used ICDS	0.346	0.351	0.342	0.009
Household used JSY	0.179	0.188	0.174	0.013
Household used JSSK	0.031	0.034	0.030	0.005

Note: 'Household used IAY', N = 2675.

***Indicates significant difference at p < 0.01, ** at p < 0.05, and * at p < 0.10.

#### 3.2.4 Social networks, mobility and confidence

Table 7 provides simple mean comparisons between SHG and non-SHG members for a range of social network and mobility outcomes (refer to Table 3 for more details on the definition of each variable).

**Table 7:** Summary statistics: social networks, appearance and mobility by SHG membership.

Variable		SHG Membership		Difference in Means
N = 2744	Full sample	Members	Non-Members	Members vs Non-Members
*Social network*				
Know at least 1/5 women**	0.776	0.836	0.739	0.097***
Part of social group with at least 1/5 women**	0.190	0.402	0.058	0.344***
Spoke to at least 1/5 women in last 5 months**	0.723	0.787	0.684	0.103***
Social network score (range: 0–1)	0.466	0.522	0.431	0.091***
Spoke with >9 people in the last 30 days in hamlet*	0.786	0.824	0.762	0.062***
Spoke with >9 people in the last 30 days nearest hamlet*	0.508	0.537	0.491	0.047
Could borrow 1000 rupees from at least 10 people within hamlet/village*	0.065	0.081	0.055	0.025**
Could borrow 1000 rupees from at least 10 people in the closest hamlet/village*	0.215	0.189	0.231	-0.042
*Mobility and confidence in public spaces*				
Does not need permission to go to at least one place*	0.252	0.263	0.245	0.018
Does not need permission to go to a village meeting or meeting of an association	0.112	0.137	0.096	0.041**
Comfortable in speaking in public*	0.230	0.273	0.202	0.071***
Mobility and confidence score (range:0–1)	0.198	0.224	0.181	0.043***

Note: 'Spoke >9 people in the last 30 days in hamlet' is a dummy variable indicating whether respondent spoke to more than 9 people in her hamlet in the last 30 days. 'Spoke >9 people in the last 30 days nearest hamlet' is a dummy variable indicating whether respondent woman spoke to more than 9 people in the nearest hamlet in the last 30 days. 'Could borrow 1000 rupees from at least 10 people within hamlet/village' is a dummy variable. 'Could borrow 1000 rupees from at least 10 people in the closest hamlet/village' is a dummy variable. '**' Social variables are constructed as dummy variables based on answering in the positive to at least 1 of the 5 randomly chosen women from the sample of 20 in the village. 'Does not need permission to go to at least one place' indicates that the respondent woman never requires permission to go to at least one of 7 places such as the market, a friend/relative's house, the mosque/church, a group meeting etc. 'Comfortable speaking in public' refers to women feeling comfortable to speak up on matters related to infrastructure, wages for public works, and misbehavior of authorities/elected officials. '# of people spoken to in 30 days, hamlet', N = 2665; '# people spoken to in 30 days, nearest hamlet', N = 2018; '# people borrow 1000 rupees, hamlet', N = 2743; '# people borrow 1000 rupees, nearest hamlet', N = 2189. ***Indicates significant difference at p < 0.01, ** at p < 0.05, and * at p < 0.10.

Each respondent was asked several questions about a random sub-sample of 5 women selected from the sample of 20 women in the same village. The respondent was asked whether she knew each of these women, and if yes, whether they were members of the same group, whether she had spoken to or exchanged information with them, and whether she would leave her child with them in case of an emergency.^12^ We find that, compared to non-members, SHG members are more likely to know at least one of the five randomly chosen women (0.84 versus 0.74, unadjusted p < 0.01). Not surprisingly, they were also more likely to be a part of a social group with some of these women (0.40 versus 0.06, unadjusted p < 0.01). When we combine all the social network questions to construct the social network score, the average score among SHG members is 0.52 and that among non-members is 0.43, reflecting the divergence in overall social networks across the two groups. We also find that compared to non-SHG women, SHG women are more likely to be able to borrow INR 1000 from at least 10 people in their hamlet/village (8.1% versus 5.5%, unadjusted p < 0.05). The borrowing network could include anyone from the hamlet/village.

Finally, regarding mobility and confidence, SHG members felt slightly more comfortable speaking in public, on average, compared to non-members, and were also less likely to require permission to go to a village meeting or meeting of an association.

## 4 Methods

This paper aims to examine the effect of SHG membership on the outcomes of interest. Although one could assess impact by comparing mean outcomes for women who are SHG members to non-members, this approach does not recognize that women who are SHG members are likely to be systematically different from nonmembers. As seen in Table 2, women who are SHG members are, on average, older and more likely to have been married longer compared to those who are not members; they are also more likely to come from better-off households. As a result, the average difference in an outcome of interest between SHG members and non-members, or the difference in unconditional means in the evaluation literature, is a biased estimate of impact; it reflects also these systematic differences between SHG members and non-members.

To eliminate this bias, we must construct a comparison group from among non-members that were similar to SHG members before the SHGs were introduced. The preferred approach to constructing such a comparison group is to randomly provide access to the program among similarly eligible individuals. But because the introduction of such SHGs was not randomly assigned across villages in our sample, this method was not feasible. The absence of “hard” targeting criteria (such as a means test, see (Pitt et al., 2006) precluded the use of Regression Discontinuity Design and, because candidate instruments for membership were weak, we decided to use matching methods. Specifically, we constructed a comparison group by matching SHG members to non-members based on observable respondent, household and community characteristics. We estimate impacts of SHG membership using nearest neighbor matching (NNM) – a form of covariate matching in which the comparison group sample of non-members is selected based on similarity to the SHG member sample in observable characteristics (Abadie et al., 2004, Abadie and Imbens, 2006).^13^

Some details and limitations of the matching procedures used deserve attention. Matching is based on variables that are associated both with the probability of being an SHG member and with the outcome of interest (Heckman & Navarro-Lozano, 2004). However, these variables should be determined before the SHGs were established to ensure that they were not affected by the SHG membership itself. Since our data comes from a single cross-section, we do not have data on these observables before the women became members. Therefore, we use variables that are either exogenous or predetermined- such as age, education and marital status of the respondent women, the caste category she belongs to, and her household’s age and gender composition. We also do not have much information on selection criteria of the SHGs that operate in these areas. As mentioned in Section 3, these are mostly organized to group women from similar socioeconomic backgrounds with the objective of economically empowering them through savings and credit activities.

**Fig. A1:**
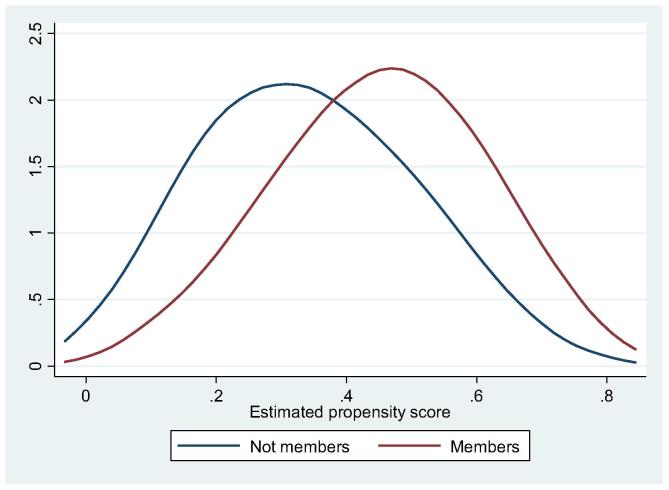
Kernel density of probability of SHG membership.

Appendix Table A1 presents the probit model of the probability that the respondent woman belongs to a SHG, as a function of individual characteristics, characteristics of the marriage, household characteristics, whether the household is in a PRADAN area, and state and district dummies. These results show that that woman’s age, women’s say in decision-making, access to multiple sources of credit (other than through the SHG) and average wealth levels in the village are important correlates of SHG membership. This model is used to compute the propensity score for the matching exercises, to check that the balancing property across the SHG members and non-members is satisfied, to ensure common support of the propensity score between the two groups (shown in Appendix Fig. A1) and to obtain a trimmed sample which excludes observations with extremely high and low propensity scores. The nearest neighbor matching model is estimated on this trimmed sample.

We use a comprehensive list of individual-level, household-level, village-level and geographic characteristics in our estimations. Individual characteristics include the age and age squared of the woman, dummy variables for primary and more than primary education (the excluded category is no schooling), and for occupation (dummies for whether she is a day laborer, and whether she is a housewife). Characteristics of the marriage include the woman’s marital status, the number of years she has been married (if married), and dummy variables for the presence of the husband at the time of interview. In addition, we control for indicators of financial resources (has own money to use, can borrow from multiple sources excluding the SHG), indicators of work load (hours spent at work, leisure hours) indicators of decision-making (participates in decisions regarding health expenses), and various indicators related to locus of control, self-esteem, and trust.

Household-level demographic variables include household size and the number of individuals in various age-sex categories, and dummy variables for the caste of the household head. The probit also includes controls for the household’s relative wealth.^14^ Finally, we control for village-level averages for landholdings, large and small livestock, village averages for women’s years of schooling, and for geographic location by including state and district dummy variables. Thus, we are effectively matching SHG members with non-members within the same broad locality, an important consideration since our data spans several culturally, economically and geographically diverse states.

In addition to presenting the matching estimates, we present the simple ordinary least squares estimates of the relation between SHG membership and the outcomes of interest as follows:

$$Y_{\mathrm{ihds}}=\alpha +\beta {\mathrm{SHG}}_{\mathrm{ihds}}+\gamma W_{\mathrm{ihds}}+\theta X_{\mathrm{hds}}+\delta_{d}+\mu_{s}+\varepsilon_{\mathrm{ihds}}$$

where $Y_{\mathrm{ihds}}$ is the outcome of interest for woman *i* in household *h* in district *d* of state *s*, $\mathrm{SH}G_{\mathrm{ihds}}$ is a dummy variable indicating whether the respondent woman is an SHG member, $W_{\mathrm{ihds}}$ is the vector of the respondent woman’s characteristics mentioned above,$X_{\mathrm{hds}}$ is a vector of household characteristics for household *h*, and $\delta_{d}$ and $\mu_{s}$ are district and state dummies respectively. Finally, $\varepsilon_{\mathrm{ihds}}$ is the individual-specific error term clustered at the block level.

## 5 Results

We first examine the association between SHG membership and political participation, awareness, and utilization of government entitlement schemes. We then explore potential mechanisms through which SHG membership could affect these outcomes. The mechanisms explored are social networks and mobility.^15^

### 5.1 Political participation

Increasing political awareness is one of the key programmatic features of many, although not all, SHGs. Not surprisingly, both OLS and NNM estimates indicate a positive significant association of SHG membership with various indicators of political participation. NNM estimates show that, compared to non-SHG members, SHG members are more likely to have voted in the last election (p < 0.01), to have voted because it was their right (p < 0.01), and to have made the decision of who to vote for on their own (p < 0.01), and, have an overall political participation score that is higher (p < 0.01) (Table 8). SHG members were also more likely to have attended a meeting of adult women in the village (*mahila gram sabha*) as well as the *gram sabha*, which involves both male and female adults, and to believe that the local governing body, the *gram panchayat*, will take positive action to demands/suggestions made by women and the SHG.

**Table 8:** Political participation.

	Respondent woman voted in the last election	Respondent woman voted because it is her right to vote	Respondent woman voted and made this decision herself	Respondent woman has ever participated in *mahila gram sabha*	Respondent woman has ever participated in *gram sabha*	Respondent woman believes that the *gram panchayat* will take positive action to demands/suggestions made by woman/SHG	Political participation score
	(1)	(2)	(3)	(4)	(5)	(6)	(7)
**PANEL A**							
OLS	0.058***	0.058***	0.053**	0.107***	0.079***	0.094***	0.075***
	(0.009)	(0.015)	(0.021)	(0.019)	(0.014)	(0.033)	(0.010)
Observations	2,733	2,733	2,733	2,733	2,733	2,733	2,733
R-squared	0.262	0.160	0.207	0.101	0.087	0.129	0.228
**PANEL B**							
NNM	0.041***	0.072***	0.071***	0.098***	0.065***	0.089***	0.073***
	(0.012)	(0.019)	(0.022)	(0.015)	(0.012)	(0.031)	(0.009)
Observations	2719	2719	2719	2719	2719	2719	2719
Mean	0.867	0.260	0.390	0.092	0.067	−0.412	0.305

Notes: ***Indicates statistical significance at p < 0.01, ** at p < 0.05, and * at p < 0.10.

### 5.2 Awareness and use of government entitlements

Finally, Table 9, Table 10 present OLS and NNM estimates of the impact of SHG membership on awareness and use of government entitlement programs. OLS estimates suggest that SHG membership is positively associated with awareness only of MGNREGA, IAY and JSY, and does not show a significant association with awareness of the other public entitlement schemes (Table 9). In contrast, the NNM estimates suggest that SHG membership increases awareness of MGNREGA only (p < 0.01).

**Table 9:** Awareness of public entitlement schemes.

	Aware MGNREGA	Aware IAY	Aware PDS	Aware AYY	Aware ICDS	Aware JSY	Aware JSSK
	(1)	(2)	(3)	(4)	(5)	(6)	(7)
**PANEL A**							
OLS	0.070***	0.045**	0.020	0.023	0.004	0.047*	−0.011
	(0.016)	(0.021)	(0.015)	(0.015)	(0.018)	(0.023)	(0.014)
Observations	2,733	2,664	2,733	2,733	2,733	2,733	2,733
R-squared	0.203	0.170	0.129	0.108	0.199	0.192	0.128
**PANEL B**							
NNM	0.056***	0.036	0.008	0.027	−0.003	0.036	−0.017
	(0.019)	(0.022)	(0.019)	(0.023)	(0.021)	(0.023)	(0.011)
Observations	2719	2650	2719	2719	2719	2719	2719
Mean	0.736	0.660	0.783	0.417	0.656	0.544	0.082

Notes: ***Indicates statistical significance at p < 0.01, ** at p < 0.05, and * at p < 0.10.

**Table 10:** Utilization of public entitlement schemes.

	Used MGNREGA	Used IAY	Used PDS	Used AYY	Used ICDS	Used JSY	Used JSSK
	(1)	(2)	(3)	(4)	(5)	(6)	(7)
**PANEL A**							
OLS	0.060***	0.031	0.043**	0.036**	0.013	0.034**	−0.002
	(0.019)	(0.019)	(0.015)	(0.015)	(0.016)	(0.014)	(0.010)
Observations	2,733	2,664	2,733	2,733	2,733	2,733	2,733
R-squared	0.184	0.087	0.319	0.060	0.252	0.213	0.146
**PANEL B**							
NNM	0.041*	0.032*	0.012	0.022	−0.012	0.026*	−0.009
	(0.022)	(0.017)	(0.023)	(0.018)	(0.021)	(0.016)	(0.007)
Observations	2719	2650	2719	2719	2719	2719	2719
Mean	0.457	0.145	0.479	0.162	0.346	0.179	0.031

Notes: ***Indicates statistical significance at p < 0.01, ** at p < 0.05, and * at p < 0.10.

Why might SHG membership not increase awareness of more entitlement schemes? We propose the following explanations. The first possibility is that NGOs working to improve knowledge around entitlement schemes focus their energies on those that are available to the majority of group members, e.g. household-level schemes like the PDS and MGNREGA, rather than those limited to a specific demographic category within those households. Indeed, convergence of the MGNREGA with the NRLM has been pushed by activists and government officials since the inception of the workfare scheme, with SHG women being mobilized to audit the scheme, report irregularities, and in some cases even to maintain the muster rolls. Second, several of the schemes outlined in Table 1 have been part of the policy landscape for many years - ICDS, for example, was introduced in 1975, and the PDS (in a different form) in 1947—whereas others were introduced relatively recently (JSSK only dates back to 2011 and JSY to 2005). Thirdly, although the descriptive statistics in Table 5 suggest that SHG members are significantly more likely to know about these entitlement programs, awareness may be correlated with factors that determine membership. Finally, as Table 1 highlights, several schemes are targeted at women within the 1000-day window, and so may not be relevant to SHG members, who are typically older. Only 4.8% of our respondents were pregnant at the time of the survey, and less than 40% had a child under the age of 5. It is plausible that recipients do not retain much information about schemes that are not deemed immediately relevant to their situation.

SHG membership, however, significantly increases utilization of government entitlements (Table 10). OLS estimates show that a respondent woman who belongs to an SHG is significantly more likely to have availed of MGNREGA, PDS, AAY and JSY. NNM estimates indicate a similar trend although only a few of the coefficients are significant: MGNREGA, IAY and JSY (p < 0.1). As mentioned above, the push for the convergence of NRLM and MGNREGA might be responsible for the increased participation of SHG women in the latter. Working on MGNREGA sites requires the woman to leave the home and interact with other men and women from the community, so the increased mobility and self-confidence of SHG women is crucial to her ability to avail of this public entitlement scheme. SHG women are also significantly more likely to have bank accounts, a prerequisite for receipt of wage payments through the MGNREGA. Finally, accessing the IAY requires the beneficiary to pay the up-front costs for the house, and be reimbursed at a later date. SHG women may have an advantage in being able to access loans more easily and at a lower cost.

### 5.3 Social capital

Table 11 presents OLS and nearest-neighbor matching estimates of the impact of SHG participation on various measures of social capital. OLS estimates show that membership is positively associated with the probability that the respondent woman knows at least one out of the five randomly selected sub-sample of women she is asked about (p < 0.05), is part of a social group with at least one of these five women (p < 0.01), and has spoken to at least one of the five women in the last six months (p < 0.05). When SHG members are matched with similar non-members, we discern similar effects of membership (p < 0.01), with the respondent woman being more likely to know at least one of the five women, to be part of a social group with them, and to have spoken with at least one of them in the last six months if she is an SHG member. These results are expected, given the modality of self-help groups and the way that they are organized.

**Table 11:** Social networks within and outside the hamlet.

	Know at least 1/5 women	Part of social group with at least 1/5 women	Spoke to at least 1/5 women in last 5 months	Spoke to >9 people in the last 30 days in hamlet	Spoke to >9 people in the last 30 days nearest hamlet	Could borrow 1000 rupees from at least 10 people within hamlet/village	Could borrow 1000 rupees from at least 10 people in the closest hamlet/village	Social networks score
	(1)	(2)	(3)	(4)	(5)	(6)	(7)	(8)
**PANEL A**								
OLS	0.054***	0.316***	0.058***	0.029*	0.012	0.597***	−0.029	0.066***
	(0.017)	(0.022)	(0.019)	(0.014)	(0.025)	(0.185)	(0.022)	(0.009)
Observations	2,733	2,733	2,733	2,733	2,733	2,732	2,733	2,733
R-squared	0.121	0.245	0.117	0.116	0.152	0.095	0.400	0.227
**PANEL B**								
NNM	0.054***	0.323***	0.056***	0.028	0.014	0.395*	−0.019	0.067***
	(0.019)	(0.018)	(0.021)	(0.017)	(0.023)	(0.224)	(0.016)	(0.009)
Observations	2719	2719	2719	2719	2719	2718	2719	2719
Mean	0.776	0.190	0.723	0.786	0.508	0.065	0.215	0.466

Notes: ***Indicates statistical significance at p < 0.01, ** at p < 0.05, and * at p < 0.10.

Connectedness to women within a group does not necessarily mean that the respondent will expand her social circle, however, or the number of people to whom she can turn for financial assistance. OLS estimates show a positive association of SHG membership with whether the respondent woman had a conversation with more than 9 people in her own hamlet in the last 30 days (p < 0.10), and whether she could borrow INR 1000 from at least 10 people from her own hamlet (p < 0.01), however, the latter effect is less significant when matching methods are used (Table 11 Panel B). Surprisingly, NNM estimates suggest that membership is negatively associated with the respondent woman’s ability to borrow INR 1000 from at least 10 people in the nearest hamlet (p < 0.1). One possible explanation for this is that SHG members no longer need to seek financial support from outside their own hamlet and are more likely to borrow from within their own SHG (which is almost always comprised of members from within the same hamlet).

Other factors may also affect the woman’s ability to interact socially with other women, such as their husbands’ willingness to allow them to leave the homestead to attend meetings. OLS estimates suggest that SHG membership is positively associated with the probability that the woman does not need permission from her husband and/or other household member to go to a village meeting or meeting of an association they are a member of (including the SHG) (the NNM estimates are not significant) (Table 12). Both OLS and NNM estimates suggest that SHG members are more comfortable speaking in public and have an overall mobility and confidence score that is higher than non-members.

**Table 12:** Mobility and confidence in public spaces.

	Does not need permission to go to at least one place	Does not need permission to go to a village meeting or meeting of an association they are a member of (inc. SHG)	Comfortable speaking in public	Mobility and confidence score
	(3)	(4)	(5)	(6)
**PANEL A**				
OLS	0.012	0.031**	0.043**	0.028**
	(0.019)	(0.013)	(0.016)	(0.010)
Observations	2,733	2,733	2,733	2,733
R-squared	0.148	0.149	0.161	0.170
**PANEL B**				
NNM	0.014	0.021	0.053***	0.029**
	(0.020)	(0.014)	(0.019)	(0.012)
Observations	2719	2719	2719	2719
Mean	0.014	0.021	0.230	0.198

Notes: ***Indicates statistical significance at p < 0.01, ** at p < 0.05, and * at p < 0.10.

## 6 Discussion and concluding comments

Our findings show a strong positive association of SHG membership with several political participation indicators. Women who are SHG members are more likely to have voted in the last election, and to have decided to do so of their own accord and because they feel it is their right to vote. Being an SHG member also makes these women more likely to attend the *gram sabha* and to believe that the *gram panchayat* would take positive action in response to suggestions made by women and/or the SHG. This last result indicates not only trust but also confidence in women’s collective power.

In terms of knowledge of different entitlement schemes, we find that SHG members are more likely to have heard about the workfare scheme, MGNREGA, and the housing scheme, IAY but not others. However, despite similar knowledge about entitlements schemes between SHG members and non-members, we find that SHG members are more likely to utilize some of these schemes, for example MGNREGA, AAY and IAY, indicating that SHG members may be more able to translate their information into action, either because of their individual empowerment (e.g. mobility), or because of the strength of the collective (e.g. engaging the SHG in social audits).

Our findings also show that membership in a SHG has a positive effect on several social network outcomes – with SHG women being more likely to know other women in their village, be part of a social group with them and to talk to them about important matters like health and nutrition. We find that SHG women are slightly more likely to be able to borrow money from someone from a neighboring village, indicating that the social network effect goes beyond the village they live in. Furthermore, SHG members are less likely to need permission from their husbands or other household member to go to a village meeting – indicating improved ability and self-confidence in interacting with those outside their household.

Going back to the pathways and the constraints identified above – information and ability to hold public entities accountable – and the channels through which SHG membership may alleviate them, our findings confirm the existence of the cross cutting SHG pathways (identified in Kumar et al., 2017) – building social capital (improved social networks) and promoting women’s empowerment (increased confidence as measured by the comfort in public speaking variable and increased mobility). They also indicate that these factors culminate in increased political participation. Our findings suggest that the information about public entitlements is not widespread despite the positive effects on social networks, self-confidence and mobility. This emphasizes the need for more focused delivery of this information through SHGs, which would then trigger the “rights pathway” (Kumar et al., 2017). Our findings show that SHGs have the potential to increase their members’ ability to hold public entities accountable and demand what is rightfully theirs. An important insight, however, is that the SHGs themselves cannot be expected to increase knowledge of public entitlement schemes in the absence of a deliberate effort to do so by an external agency.

Our results are consistent with a growing body of qualitative evidence on how self-help groups contribute to collective social behavior, participatory democracy and governance in India (Rao and Sanyal, 2010, Sanyal et al., 2015, Sanyal et al., 2015). SHG members have larger social networks and participate more actively in their local democratic bodies. Increased confidence may also come from exposure to associational life, a consequence of belonging to an SHG. In an analysis of 255 *gram sabha* transcripts, (Sanyal, Rao, and Prabhakar (2015)) found that women associated with microcredit SHGs have a higher quality of participation in meetings, not because they talk more often, or raise more issues, but because they are able to present the context for the problem, use a public goods framing, and show awareness that *panchayat* and government officials were accountable.

Greater awareness and utilization of public entitlement schemes among SHG women, as observed in our results, could be a result of the women taking up issues for which they have found common ground. This is consistent with (Sanyal, Rao, and Prabhakar (2015))’s finding that women SHG members participating in *gram sabhas* frame their narratives in terms of common issues. Overall, our results indicate the potential for SHGs to empower women both individually and collectively, which may lead to better awareness, accountability, and governance of public entitlement schemes.

## Conflict of interest statement

The authors of this article wish to confirm that there are no known conflicts of interest associated with this publication. None of the coauthors has received financial support for this work that could have influenced its outcome.

We also confirm that the manuscript has been approved for submission by all seven co-authors, and that all co-authors have approved the order of the listing of the authors. There is no person who contributed significantly to this manuscript but has not been listed as a co-author.

## Figures and Tables

**Table A1:** Probit of the propensity score estimation.

Variables	Probability of being an SHG member
Age of women respondent	0.146***
	(0.033)
Squared age of women respondent	−0.002***
	(0.000)
Respondent woman has 1–5 years of schooling	0.115
	(0.077)
Respondent woman has more than 5 years of schooling	0.153*
	(0.080)
Ag & Non-Ag day Laborer	−0.019
	(0.064)
Housewife	−0.108
	(0.077)
Caste of household head, SC	−0.115
	(0.147)
Caste of household head, ST	−0.183
	(0.136)
Caste of household head, OBC	0.023
	(0.141)
Married	0.163
	(0.150)
# years married+	0.021**
	(0.009)
Dummy for whether the husband of respondent woman is present in HH	−0.054
	(0.128)
Has own money to use	0.095*
	(0.056)
Talk often to own family other than HH	−0.037
	(0.056)
Leisure hours per day	−0.002
	(0.008)
Work hours per day	0.014
	(0.008)
# of children under 5 years	0.036
	(0.053)
No. females age 10–55 years	0.013
	(0.068)
Ability to borrow from multiple sources	0.132*
	(0.075)
Social status weight	0.256
	(0.349)
Sum of 4 locus of control questions (range 4–16)	0.018
	(0.016)
sum of 4 esteem questions (range 4–16)	−0.017
	(0.016)
sum of 4 trust questions (range 4–16)	0.007
	0.079**
Log of Total monthly consumption expenditure, per capita	(0.035)
	−0.141*
Poorest wealth quintile	(0.080)
	−0.021
HH owns more land than average in that district	(0.059)
	0.021
HH owns more large livestock than average in that district	(0.060)
	0.032
HH owns more small livestock than average in that district	(0.070)
	0.012
Women's average education per village	(0.026)
	−0.054**
Range of highest and lowest wealth index in village	(0.024)
	0.082**
Average land owned by HH in village (acres)	(0.032)
	0.016
Avg. number of large livestock owned by HH in village	(0.033)
	0.132***
Avg. number of small livestock owned by HH in village	(0.035)
	0.079**
Observations	2,733

Notes: Also included in the probit are variables reflecting age and sex composition of the household, state and district dummies. Standard errors in parentheses. ***p < 0.01, **p < 0.05, *p < 0.1.

## Appendix

Table A1 and Fig. A1.

## Footnotes

1 There are other constraining factors, such as the supply side and the coordination between supply and demand. We will touch upon the coordination of supply and demand but mostly focus on the demand side factors in this paper. The supply side constraints include the inability of the responsible public entities to identify the potential beneficiaries and reach them in a cost-effective way, divergence between financial allocations and ground realities of total eligible beneficiaries.

2 One element of accountability that we do not address in this paper is the accountability of the organizing NGO to its members. Kilby (2011), based on case studies of 15 NGOs as well as in-depth interviews with 80 women’s self-help groups in southern India, finds a strong correlation between empowerment and those NGOs with strong ‘downward’ accountability mechanisms. These findings support the notion that empowerment within women’s lives, particularly in terms of ‘agency’, was stronger if the women in the self-help groups had a direct role in some of the institutional processes of the organization that facilitated that change (in this case the NGOs). The data that are available to us at this point preclude the analysis of this issue, which can be addressed in the ongoing process evaluation of the SHG platform.

3 The question posed did not specify what level the elections were held at, so this could be an election at any level (general election, *panchayat* election etc).

4 One could argue that political participation may be an ambiguous measure of empowerment because empowered persons can also choose not to participate. For example, sex workers, even if “empowered”, may choose not to vote given how their work is marginalized in the political process. Nevertheless, a large number of studies do find positive impacts of women’s groups on some measures of political participation, as discussed above.

5 Please refer to the following for greater details on these programs: NRLM (http://www.worldbank.org/en/news/feature/2011/07/05/the-national-rural-livelihoods-project), SERP (http://healthmarketinnovations.org/program/society-elimination-rural-poverty-serp-indira-kranthi-patham), JEEViKA (http://projects.worldbank.org/P090764/bihar-rural-livelihoods-project-jeevika?lang=en&tab=overview).

6 At present, $1 is approximately equal to INR 65.

7 For further details, see http://aajeevika.gov.in/content/state-missions.

8 The respondent women were aware of the names (and occasionally positions) of the individuals from the organization that they interact with, but were unable to answer the (unprompted) question ‘Which organization supports this self-help group?’. We believe that this relates more to the way the question was phrased than to a lack of awareness of the NGO/GO actually providing support, because if prompted, in most cases names of the organization in question were recognized.

9 In response to the question “Does *gram panchayat* take action to address a complaint or suggestion raised collectively by women/SHGs?”, only 3% of the women responded “Yes, always”, and only 7.6% said “Yes, sometimes”, as compared to 51.8% who said “never”. So, the low attendance in the *gram sabha* and *mahila gram sabha* could also reflect lack of confidence in the political process.

10 Since we rely on self-reporting for whether the women voted independently, it is plausible that the level of family interference is underestimated. There are a series of questions in our survey to get at this. For example, we ask the respondent women reasons for not voting at all. The primary reason cited is “not having the voter card”. About 4% were fearful or were stopped by husband. We also asked them reasons for voting and the distribution of responses is the following: husband asked (19%), my right (30%), political party either gave incentive or requested for the vote (10%), in anticipation of help (30%), for conformity with others (9%). When posed the question, “In your house, who decides whether you vote?”, the responses given were: Self (40.3%), husband (36.4%), elder family member (7.6%), village level influential (7.9%), others (2.2%) and don’t know (5.6%). This shows that a sizeable number of respondents did in fact mention that they did not take the decision themselves. The variables used to indicate “voted because it is her right to vote”, “voted and made the decision herself” are based on a combination of responses to these questions (see Table 3). We should also clarify that the husband or other adult family members were not present during the interview, which somewhat alleviates concerns of responses being influenced.

11 We acknowledge that low utilization of government schemes could be a result of a failed political process and limited accountability of government officials, which could cause low knowledge as well as low supply of these services (and hence to low utilization rates).

12 Because each woman was asked only about a subset of other women from the same village, we were unable to construct reciprocal links, so all measures of social networks are self-reported. In the literature on social networks, such ‘out-degree’ measures of social ties are considered less reliable than ‘in-degree’ measures, where others report having a link with the individual in question. As a result, the reader should note that the possibility that our measures overestimate network size.

13 These approaches rely on two assumptions about the data and the model. The first is that, after controlling for all pre-program observable respondent, household and community characteristics that are correlated with program participation and the outcome variable, non-beneficiaries have the same average outcome as beneficiaries would have had if they did not receive the program. The second assumption is that for each beneficiary household and for all observable characteristics, a comparison group of non-beneficiaries with similar observable variables exists.

14 Indicators of household wealth include whether the household is in the lowest wealth quintile, whether landholdings are larger than the district average, and whether large and small livestock holdings are larger than the district average.

15 While we argue that social networks and increased mobility may be potential mechanisms that lead to increased political participation, awareness, and utilization of schemes, we do not validate that these are in fact the mechanisms through which political participation and/or awareness and utilization of government entitlement schemes increases.
